# Investigation of the mechanical properties of pineapple leaf fibre-reinforced biocomposites

**DOI:** 10.1038/s41598-025-12044-0

**Published:** 2025-08-13

**Authors:** Susai Manickam Paulsingarayar, Seenivasan Soundararajan, P. Satishkumar, Jayant Giri, T. Sathish, Muhammad Imam Ammarullah

**Affiliations:** 1 Department of Mechanical Engineering, NPR College of Engineering and Technology, Natham, 624401 Tamil Nadu India; 2 Department of Mechanical Engineering, Rathinam Technical Campus, Coimbatore, 641021 Tamil Nadu India; 3 Department of Mechanical Engineering, Yeshwantrao Chavan College of Engineering, Nagpur, 441110 Maharashtra India; 4 Division of Research and Development, Lovely Professional University, Phagwara, 144411 Punjab India; 5 Centre for Research Impact and Outcome, Chitkara University Institute of Engineering and Technology, Chitkara University, Rajpura, 140401, Punjab India; 6 Department of Mechanical Engineering, Saveetha School of Engineering, Saveetha Institute of Medical and Technical Sciences (SIMATS), Saveetha University, Chennai, 602105 Tamil Nadu India; 7 Department of Mechanical Engineering, Faculty of Engineering, Universitas Diponegoro, Semarang, 50275 Central Java Indonesia; 8 Undip Biomechanics Engineering & Research Centre (UBM-ERC), Universitas Diponegoro, Semarang, 50275 Central Java Indonesia

**Keywords:** Pineapple leaf fibre, Biocomposite tensile, Compression, X-ray spectroscopy (EDX), Engineering, Mechanical engineering

## Abstract

This study explores the production process and mechanical properties of biocomposites reinforced with pineapple leaf fibres. The biocomposite fabrication involves the use of various techniques, including compression moulding, to integrate the natural fibres into a polyester matrix. The mechanical performance of the resulting composites is evaluated through a comprehensive set of tests, including tensile strength measurements and Energy Dispersive X-Ray Spectroscopy (EDX). Key factors influencing mechanical behaviour, such as fibre content, orientation, and production variables, are systematically examined. Additionally, the chemical composition of the composites is assessed to understand its role in their performance. The findings provide valuable insights into the potential applications of pineapple leaf fibre-based biocomposites in industries requiring lightweight, sustainable materials with enhanced mechanical properties. This paper aims to establish a clear link between production techniques and mechanical performance, highlighting the practical potential of these biocomposites for environmentally-conscious material solutions.

## Introduction

Composites are advanced materials that consist of two or more distinct phases, combining their unique properties to yield a superior material with enhanced performance. These materials typically comprise a matrix, which is usually made from polymers, metals, or ceramics that binds reinforcing phases such as fibers, particles, or flakes. The resulting composite material benefits from the synergistic properties of its constituents, including improved mechanical, thermal, and chemical performance^1^. Composites are well-regarded for their high strength-to-weight ratio, durability, and ability to withstand extreme conditions, making them essential in industries such as aerospace, automotive, construction, and sports^2^.

In recent years, there has been a growing shift towards the development of eco-friendly composites in response to escalating environmental concerns and the need to minimize reliance on non-renewable resources. This shift has fostered the rise of natural fiber composites (NFCs), which provide sustainable alternatives by leveraging biodegradable and renewable resources. NFCs offer several environmental advantages, including biodegradability, reduced carbon footprints, and the utilization of renewable materials, in contrast to synthetic fiber composites^3^. Among the many natural fibers being explored, pineapple leaf fiber (PALF) has gained significant attention due to its unique properties and its potential applications in biocomposite development^4^.

PALF is a low-cost, abundant, and renewable source of fiber, typically a by-product of pineapple cultivation, making it an attractive material for composite production. This fiber boasts high cellulose content, which imparts rigidity and tensile strength, as well as a low microfibrillar angle that enhances its strength and flexibility^5^. Furthermore, PALF composites are lightweight, which is particularly advantageous for applications in sectors such as automotive and aerospace, where weight reduction is crucial^6^. The combination of these properties makes PALF an ideal candidate for use in biocomposites, offering a viable alternative to synthetic fibers like glass and carbon fibers.

Despite the promising mechanical properties of PALF-reinforced biocomposites, challenges remain in fully utilizing these materials. One of the primary issues is the hydrophilic nature of natural fibers, which leads to moisture absorption and subsequent degradation of mechanical properties over time. Additionally, achieving optimal fiber-matrix adhesion can be difficult due to the inherent incompatibility between the hydrophilic fibers and the polymer matrices^7^. Surface treatments and coupling agents are often employed to enhance fiber-matrix compatibility, but these treatments may introduce additional complexities in the processing of the composites. Moreover, variations in PALF quality due to differences in cultivation and extraction methods can result in inconsistent mechanical properties of the final composite material^8^.

To overcome these challenges, research efforts are focused on developing hybrid composites and advanced surface modification techniques to improve the mechanical properties and durability of PALF composites. These strategies aim to expand the applications of PALF biocomposites in sectors such as automotive, construction, and packaging, where environmentally friendly, lightweight, and durable materials are in high demand^9^. Moreover, the development of bio-based resins and the integration of PALF with other natural fibers are being explored to further improve the sustainability and performance of these biocomposites^10,11^. Additionally, studies have shown that PALF composites can be used in a variety of consumer goods, including furniture, packaging, and textiles, further highlighting their versatility and potential in everyday applications^12,13^. The automotive industry has also started incorporating PALF composites for non-load-bearing components, such as dashboards and seat backs, contributing to weight reduction and improved fuel efficiency in vehicles^14^. Furthermore, ongoing research into novel processing techniques, such as injection molding and compression molding, aims to optimize the performance and scalability of PALF-reinforced biocomposites for industrial-scale applications^15^.

This paper provides a comprehensive investigation into the mechanical properties of pineapple leaf fiber-reinforced biocomposites. The study examines the effects of fiber content, fiber orientation, and processing variables on the composite’s performance. Furthermore, it addresses the challenges associated with using PALF in biocomposites and highlights potential solutions, contributing to the advancement of sustainable biocomposite materials. This research, which focuses on PALF-reinforced composites, aims to promote the adoption of eco-friendly materials in various industrial applications.

## Materials and methods

### Pineapple fibre creation

**Fig. 1:**
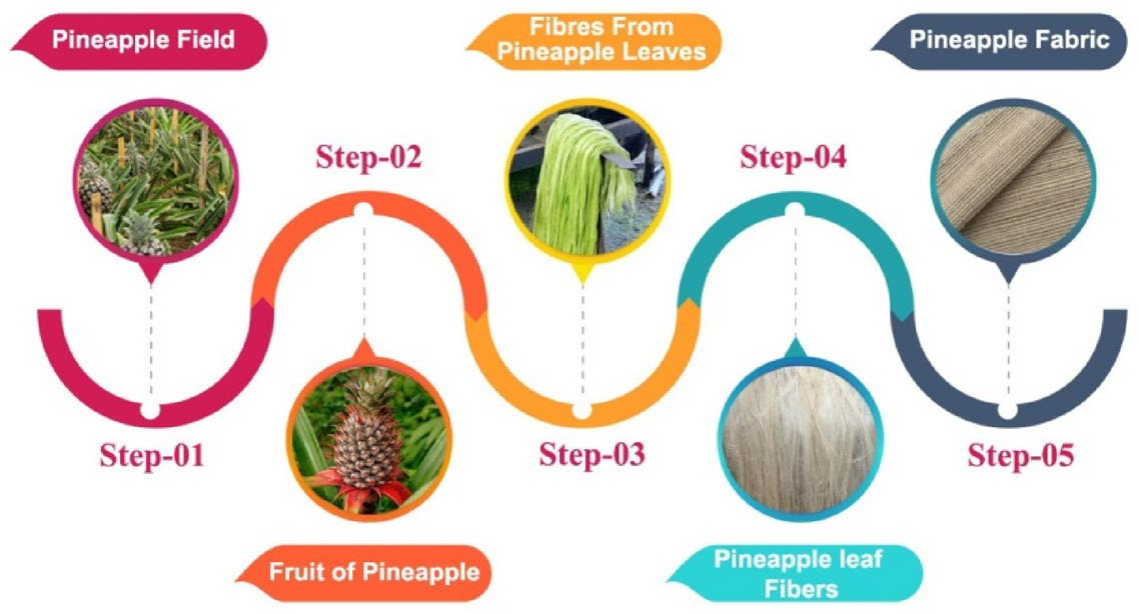
Step by step process of pineapple fibre creation.

Pineapples, perennial herbaceous plants with heights and widths of 1–2 m, are bromeliaceous. This plant is grown in coastal and tropical areas for its fruit. Pineapple production in India is rising on 2,250,000 acres. These fields support the short-stemmed, dark-green pineapple plant. The leaf emerges ornamentally and then becomes a sword-shaped structure three feet long and two to three inches wide. Spiral-shaped fibrous leaves. For stiffness, leaf edges are twisted inward. The production of pineapple leaf fiber (PALF)-reinforced biocomposites involves a systematic process (see Fig. 1) that begins with the extraction of fibers from pineapple leaves and culminates in their incorporation into a matrix material. The initial step in this process is the harvesting of mature pineapple leaves, which are typically considered agricultural waste following fruit production. Once harvested, the leaves undergo a retting process, wherein they are soaked in water or buried in the ground for a set period to facilitate the separation of the fibers from the leaf matrix. After retting, the fibers are manually or mechanically extracted from the leaves. The recovered fibers are then thoroughly washed and dried to remove any contaminants and excess moisture.

To enhance the adhesion between the pineapple fibers and the matrix material, the fibers may undergo optional surface treatments, such as chemical treatments or coatings. This step is crucial in improving the interfacial bonding between the fibers and the polymer resin. Following fiber preparation, the next phase involves the selection and preparation of the matrix material. Depending on the desired properties of the composite, a polymer resin is chosen and mixed with appropriate additives, fillers, or reinforcing agents to achieve the required composition. The composite fabrication process continues with the hand lay-up method, where the fibers are carefully arranged in a mold, ensuring uniform distribution, and then impregnated with the matrix material. This is followed by the compression molding process, in which the fiber-matrix assembly is placed into a mold and subjected to heat and pressure to form and cure the composite. Alternatively, the vacuum infusion method may be used, where the fibers are placed in the mold, and the matrix material is infused under vacuum pressure to ensure complete impregnation. This method is often employed for generating cylindrical composite structures by wrapping continuous fibers around a rotating mandrel and applying the matrix material precisely.

Once the composite has been shaped, the curing process solidifies the matrix material and firmly bonds the fibers. This curing can occur under heat and pressure or ambient conditions, depending on the resin system used. Post-processing steps, such as trimming and finishing, are then carried out to achieve the desired shape and appearance of the composite. Surface treatments may also be applied to enhance both the aesthetic and performance characteristics of the final product.

### Experimental setup

**Fig. 2:**
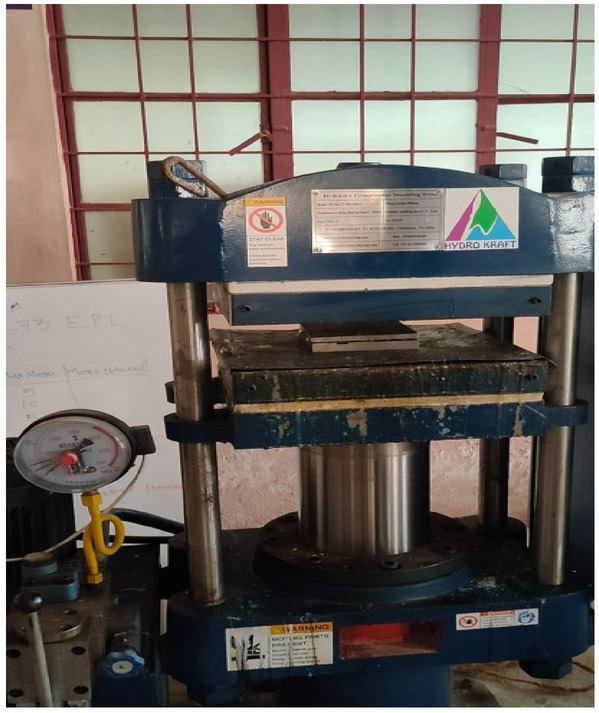
Compression moulding machine.

**Fig. 3:**
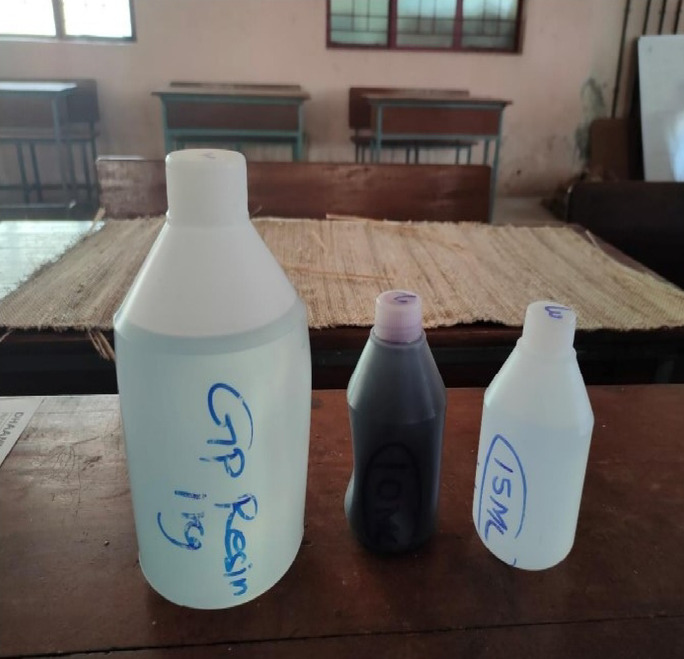
Polyester resin.

To ensure the quality of the produced composites, a series of mechanical tests, including tensile strength, flexural strength, and impact resistance, are conducted to verify that the material meets established standards. The experimental setup requires several materials and equipment, including a compression molding machine (Fig. 2), pineapple leaf fiber, unsaturated polyester resin (Fig. 3), hardener, accelerator, aluminum foil, beakers, brushes, and wax for easy removal from the molds. The resin and its hardener and accelerator are mixed in precise proportions, and the mold is coated with wax and aluminum foil to prevent adhesion of the composite to the die. The fiber is then placed alternately with the resin in the mold, and the die is subjected to heat and pressure in the compression molding machine for approximately 90 min. After cooling for an additional 120 min, the composite plates are removed from the mold. This process is repeated for varying numbers of layers depending on the desired composite efficiency, as calculated based on specific performance requirements. The relevant process parameters are summarized in Table 1.

**Table 1:** Pineapple process Parameters.

Parameters	Sample − 1 two layer	Sample − 2 four layer	Sample − 3 six layer	Sample − 4 eight layer
Pineapple fiber Vf	5%	10%	15%	20%
Polyester resin	100 g	100 g	100 g	100 g
Accelerator	5 g	5 g	5 g	5 g
Catalyst	0.5gm	0.5gm	0.5gm	0.5gm
Pressure	50 kg/cm	60 kg/cm	70 kg/cm	80 kg/cm
Temperature	130^0^c	130^0^c	130^0^c	130^0^c

### Calculation for the selection of layer

To calculate the number of layers needed for each sample, the laminate thickness, volume fraction of fiber, and fiber density were considered in determining the axial density of the fiber, which is essential for ensuring the correct composition of the composite. For Sample 1, the laminate thickness was set to 5 mm, and the volume fraction of the fiber (Vf) was 5%. The fiber density (ρf) was given as 1.3 g/cm³, or equivalently 1.3 × 10³ kg/m³. The axial density of the fiber (Af) is calculated using the Eq. (1).

1 $$Vf = \frac{{\eta {\text{~}}A_{f} }}{{\rho _{f} ~t}}.$$

Using this formula, the thickness of the laminate (t), volume fraction (Vf), and fiber density (ρf) were substituted into the equation, yielding an axial density of 160 g/m^2^ for the fiber. This value was used to calculate the number of layers (η) required to achieve the desired composite properties. The number of layers for each sample was determined as follows: For Sample 1, the calculated value of η was found to be 2 layers. Similarly, for Sample II, η equated to 4 layers; for Sample III, η resulted in 6 layers; and for Sample IV, η amounted to 8 layers.

**Fig. 4:**
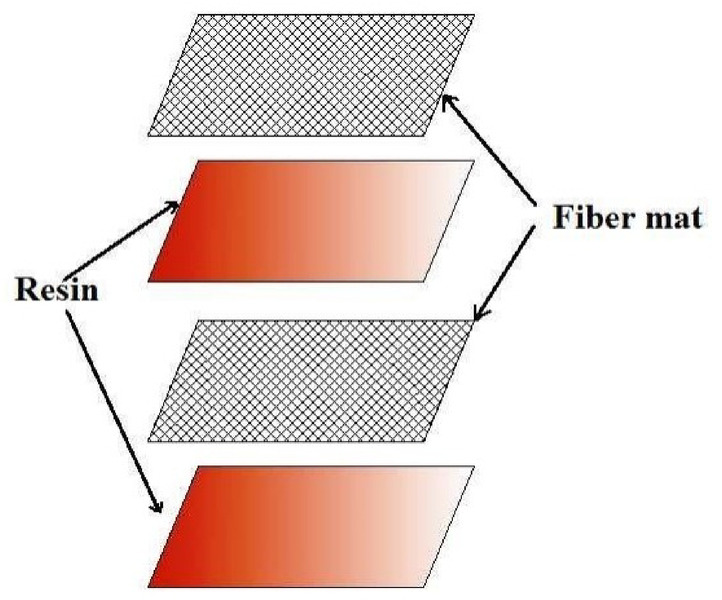
Fabrication of PALF Composite\.

**Fig. 5:**
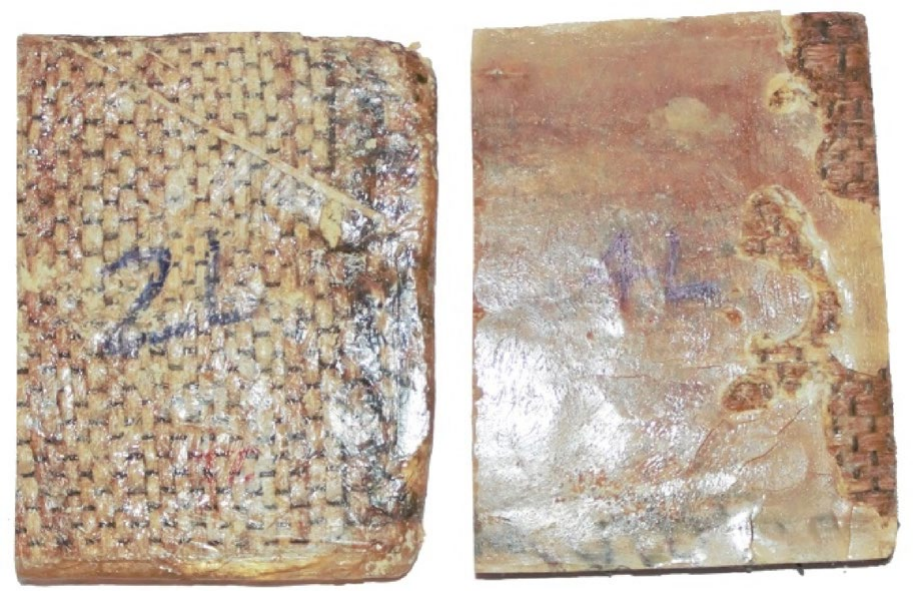
Final PALF.

**Fig. 6:**
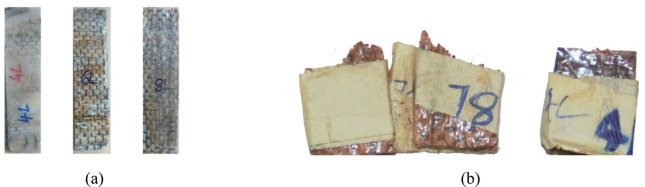
Tensile test: (**a**) before and (**b**) after.

The fabrication of the Pineapple Leaf Fiber (PALF)-reinforced composite is illustrated in Fig. 4, where the process steps are depicted. Figures 5 and 6 show the final PALF samples, with Fig. 6 specifically illustrating the samples before and after undergoing tensile testing. These figures provide a visual representation of the sample preparation, highlighting the changes in the composite structure after the mechanical testing process.

## Results and discussion

### Tensile test of PALF

The mechanical performance of Pineapple Leaf Fibre (PALF)-reinforced polymer composites was evaluated through tensile testing, following ASTM standards for specimen preparation. The specimens were subjected to testing using an Electronic Tensometer (Model PC-2000), equipped with a 20 kN load cell, operating at a crosshead speed of 5 mm/min. A gauge length of 50 mm was maintained during the tests, which were conducted at a controlled temperature of 28 °C and 50% relative humidity. During testing, the specimens were gradually loaded until failure, and key mechanical properties such as tensile strength and elongation at break were recorded. The fracture typically occurred at the midpoint of the gauge length.

**Fig. 7:**
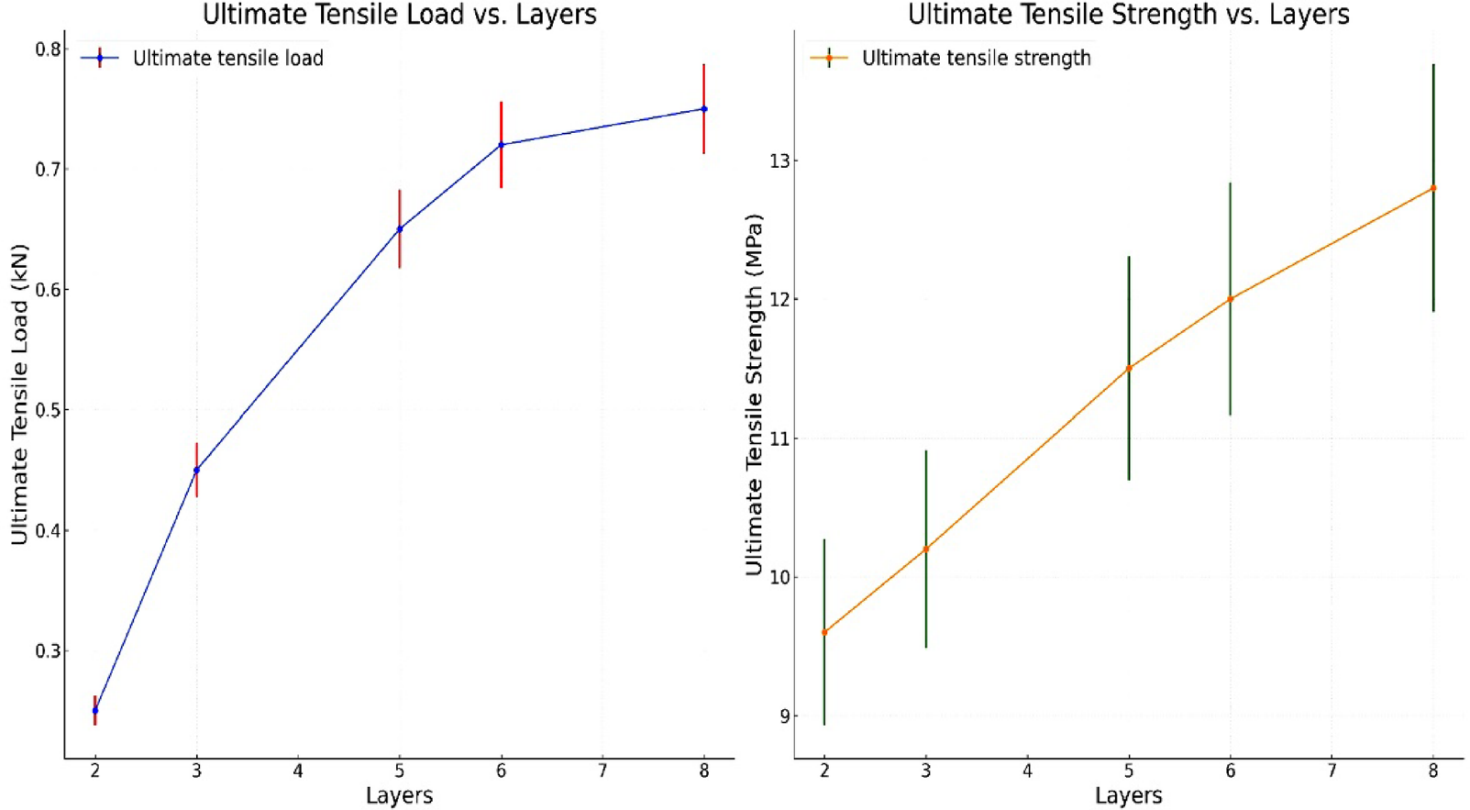
Ultimate Tensile Strength and Load Vs Layers.

Figure 7 illustrates the tensile testing results as a line graph, demonstrating a smooth and gradual transition of the stress-strain curve. This indicates consistent load transfer and uniform mechanical behavior between layers of the composite. The observed graph supports the hypothesis of a well-bonded and homogeneously distributed fiber-matrix interface, which ensures a continuous and reliable performance across the composite material. These characteristics are crucial for the accurate estimation of intermediate property values, confirming the robustness of the material under tensile stress.

From the results shown in Fig. 7, it can be inferred that the composite with a 5% volume fraction of PALF reinforcement in a polyester resin matrix exhibited an ultimate tensile strength of 9 MPa. This finding is consistent with previous research, which suggests that PALF, owing to its higher aspect ratio (AR), cellulose content, and crystallinity, can significantly improve the tensile strength of composites. However, it is important to note that the hydrophilic nature of cellulose could negatively influence the tensile properties if the fiber’s moisture content is not controlled. Additionally, the incorporation of compatibilizers into the matrix has been shown to enhance the interfacial bonding between the PALF fibers and the resin. Throgh promoting a more homogeneous fiber dispersion and reducing fiber aggregation, compatibilizers improve the mechanical strength of the composite and prevent premature failure due to fiber pull-out.

Furthermore, the uniform fiber-matrix interaction reduces the likelihood of fracture propagation within the composite, thereby enhancing the overall tensile performance. The improvements in the tensile strength and elongation at break are attributed to the effective dispersion of fibers within the matrix, which minimizes defects and stress concentrations within the material. These findings align with the results of previous studies, emphasizing the critical role of fiber treatment, matrix compatibility, and fiber orientation in determining the mechanical properties of PALF-reinforced biocomposites. The tensile testing results demonstrate that PALF-reinforced composites exhibit significant potential for use in a variety of structural applications, particularly where strength, durability, and eco-friendliness are prioritized. Further investigations into optimizing fiber treatments and resin formulations will likely yield even stronger, more versatile biocomposites.

### Elemental composition analysis using energy dispersive X-ray spectroscopy (EDS)

**Fig. 8:**
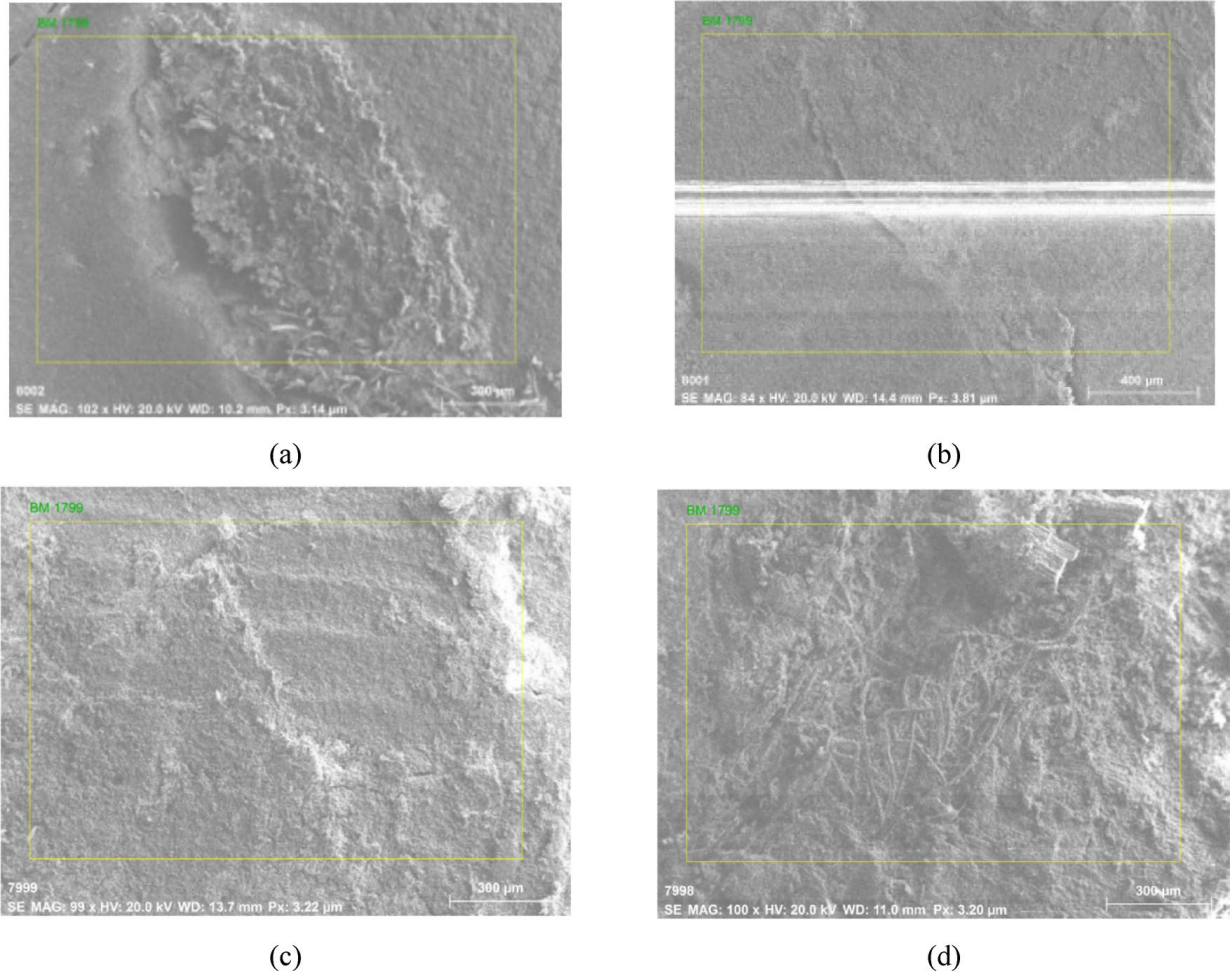
SEM results: (**a**) 2, (**b**) 4, (**c**) 6, and (**d**) 8 layers.

Energy-dispersive X-ray spectroscopy (EDS) is a highly effective analytical technique used to determine the elemental composition of materials^16^. In this method, an electron beam is directed at the sample, causing the atoms within the material to emit characteristic X-rays. These emitted X-rays are captured and analyzed by an energy-dispersive detector, generating an elemental spectrum. The peaks in the spectrum are indicative of specific elements, enabling both qualitative and quantitative analysis of the sample’s composition. EDS is extensively applied in fields such as materials science, forensics, and geology, owing to its ability to provide detailed compositional information at micron to nanoscale resolutions. This makes EDS particularly valuable for tasks like trace element identification, elemental mapping, and chemical characterization of materials. Additionally, when combined with scanning electron microscopy (SEM) ^17^, EDS enhances the ability to investigate both the elemental composition and the morphological characteristics of the sample. The SEM results for the 2, 4, 6, and 8-layer PALF composites are presented in Fig. 8a–d, respectively.

**Fig. 9:**
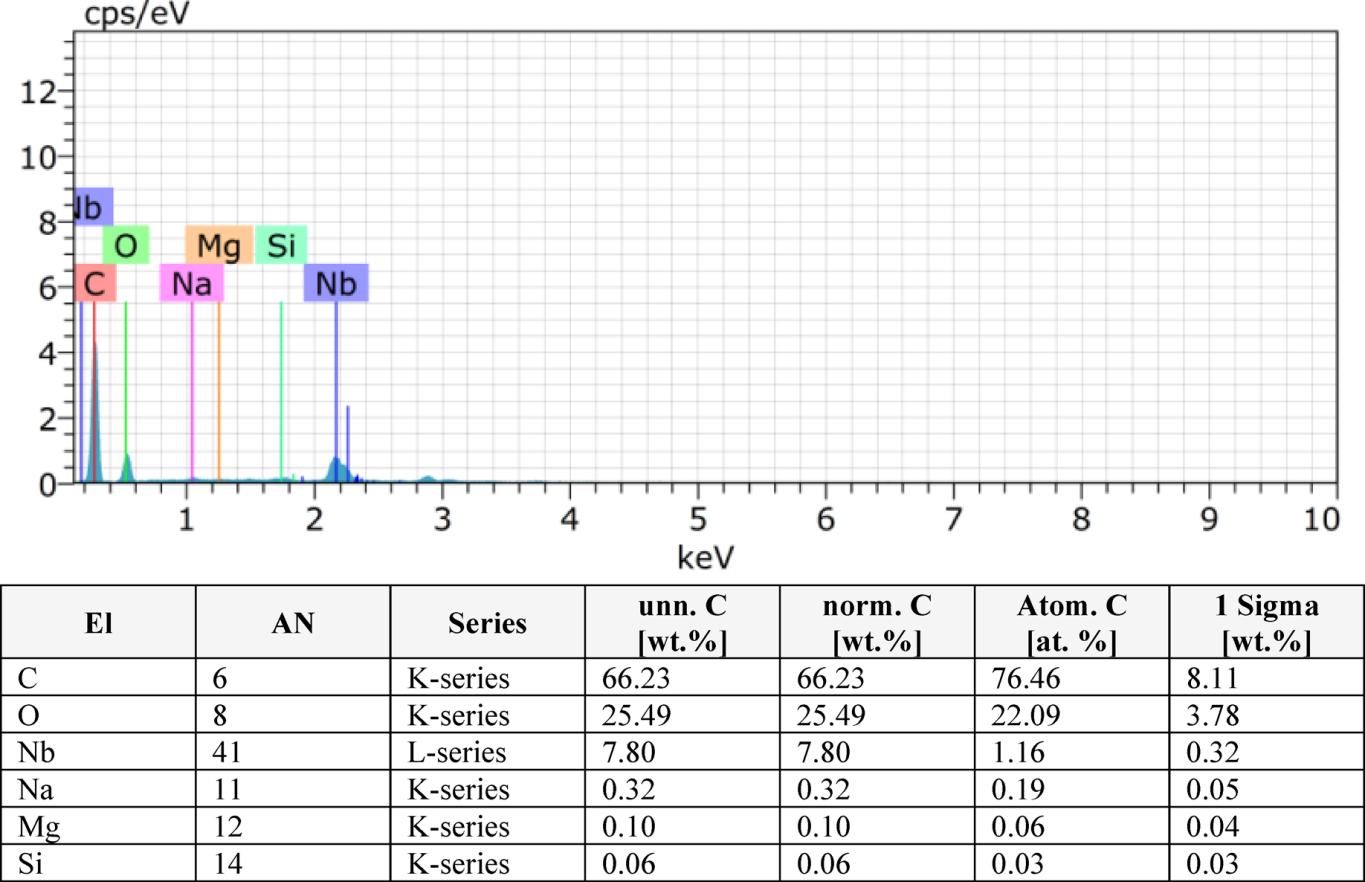
EDX result for 2 layer.

**Fig. 10:**
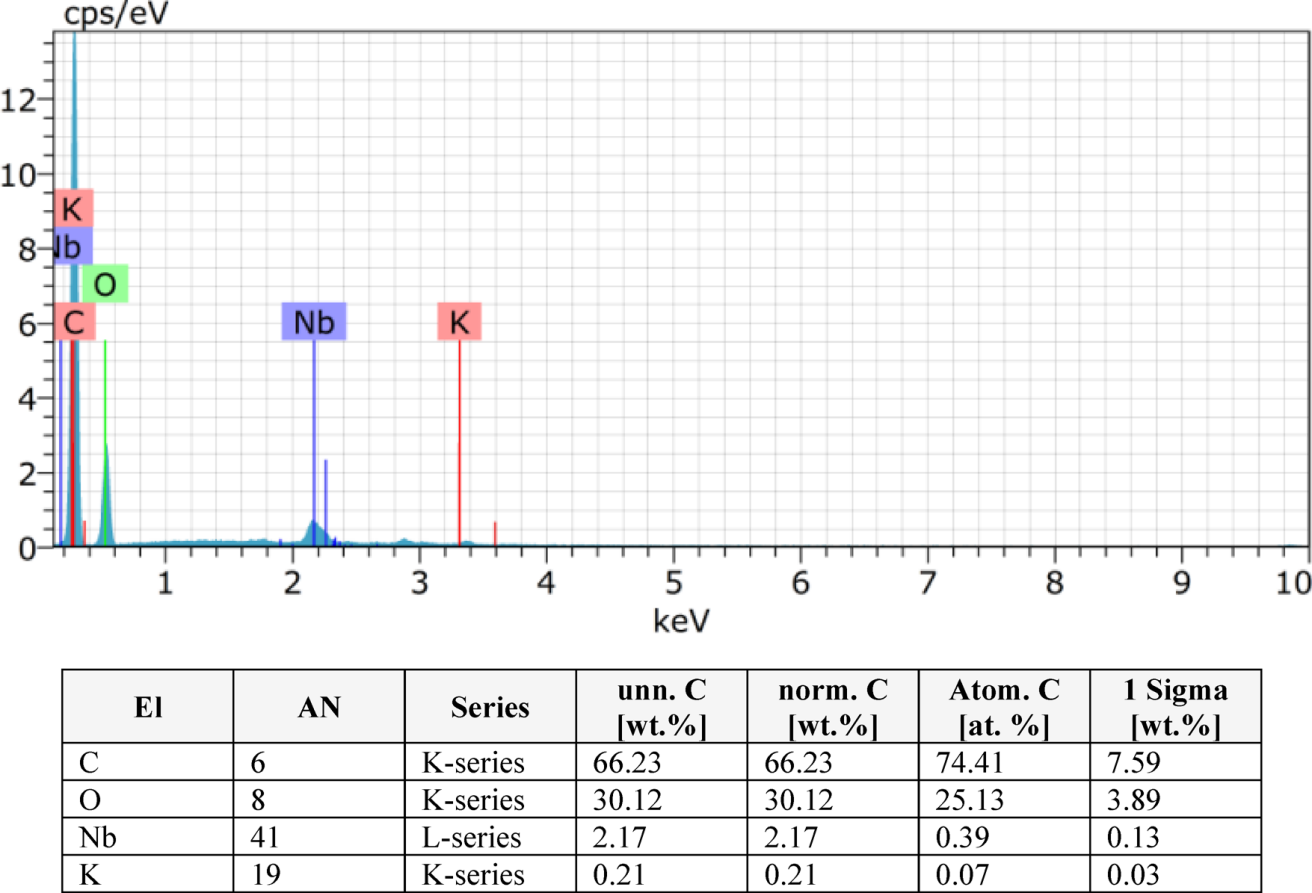
EDX result for 4 layer.

**Fig. 11:**
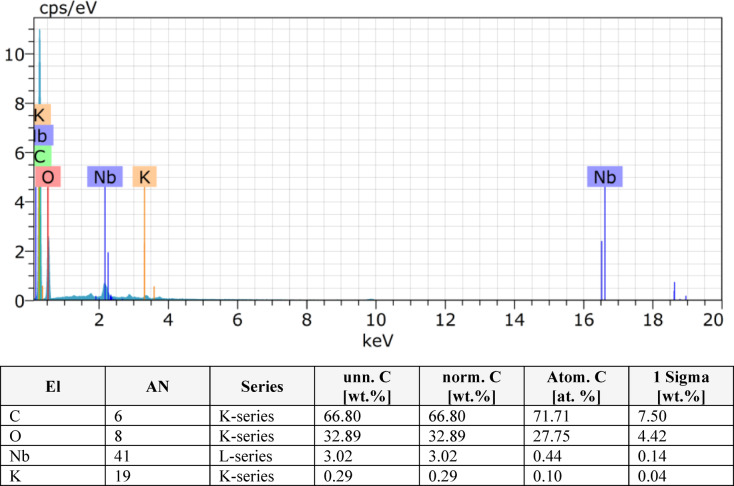
EDX result for 6 layer.

**Fig. 12:**
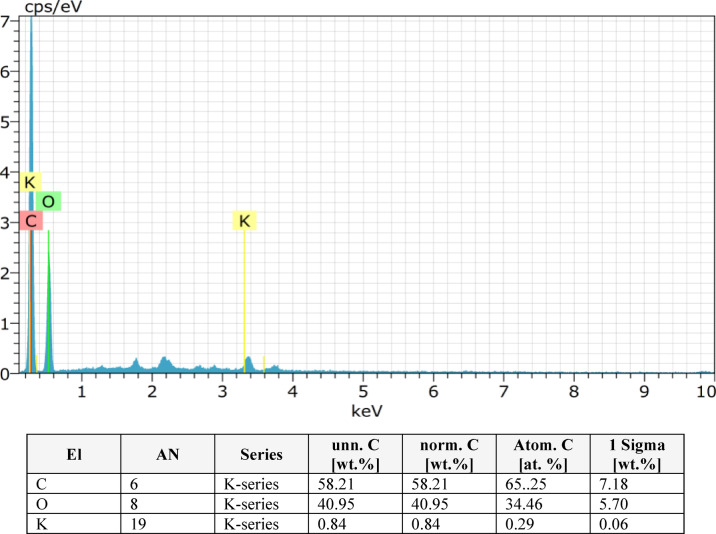
EDX result for 8 layer.

In this study, EDS was performed on pineapple leaf fibre (PALF) composites with varying layer configurations (2, 4, 6, 8 layers) as shown in Figs. 9, 10, 11 and 12. The analysis revealed that the composition of the PALF composites is predominantly carbonaceous, with oxygen as the next most abundant element. Trace elements such as sodium, silicon, niobium, and magnesium were also detected. The total elemental composition of the samples summed to 100.00 weight%, with one standard deviation (1 Sigma) accounting for the associated uncertainty in the measurements. These results highlight the carbon-rich nature of PALF composites, which may have significant implications for their potential applications in industries where carbon-based materials are highly valued. The precision in elemental characterization achieved through EDS plays a crucial role in material quality control and further aids in the development of these biocomposites for various engineering applications.

### Implications of elemental composition on the mechanical and functional properties of PALF composites

The distinctive characteristics of carbon-rich pineapple leaf fibre-reinforced biocomposites (PALF) position them as a versatile and promising material with significant potential across various applications. The high carbon content within these composites is a key factor contributing to their mechanical strength and electrical conductivity, though the specific form of carbon and its arrangement within the composite matrix are critical factors influencing performance. The examination of the elemental analysis reveals a substantial carbon content in the PALF composites, suggesting potential improvements in the mechanical properties and overall durability of the material.

With carbon constituting over 66% of the composite, the resulting material demonstrates exceptional structural strength, making it ideal for applications requiring high performance. The intrinsic rigidity and durability of carbon make it a prime candidate for reinforcement, contributing to improved tensile strength and modulus. As such, carbon-rich PALF composites possess characteristics that render them well-suited for structural applications where both lightness and strength are paramount. Examples of such applications include aerospace components, automotive parts, and biomedical equipment, where high-performance materials are essential.

Moreover, the presence of oxygen and trace elements such as niobium and potassium adds further potential to the composite’s capabilities. Oxygen, as a functional component, may enhance the material’s compatibility with specific polymer matrices, thereby improving the bonding at the interface between the fibre and the matrix, ultimately leading to improved mechanical properties. The inclusion of niobium and potassium can also enhance specific performance traits, such as corrosion resistance and catalytic activity. These features open up additional possibilities for the use of PALF composites in industries such as chemical engineering, electronics, and environmental remediation. The high carbon content of PALF composites presents a significant opportunity for the development of advanced materials with tailored characteristics. This makes them particularly appealing for diverse industrial applications that prioritize strength, durability, and performance. The unique combination of mechanical and functional properties makes PALF composites a promising material for a wide array of applications requiring both structural integrity and specialized performance. Also, the carbon content not only carbon content alone does not directly dictate strength. Fiber orientation, matrix adhesion, and dispersion also play a crucial role in defining mechanical properties.

## Conclusion

The present investigation demonstrates the successful fabrication of pineapple leaf fiber (PALF)-reinforced biocomposites using the hand lay-up method, highlighting the significant enhancement of mechanical properties such as tensile strength and Young’s modulus with increased fiber content. The study emphasizes the importance of fiber treatment, diameter, and moisture content in optimizing the composite’s mechanical performance. Elemental analysis through EDAX reveals that PALF’s high cellulose content, low mineral composition, and presence of surface hydroxyl groups contribute to its superior mechanical properties and compatibility with polymer matrices. The synergistic effects between PALF and the polyester matrix result in composites with improved structural integrity, and electrical conductivity, making them suitable for a variety of applications in automotive, aerospace, and electronics sectors. These findings suggest that PALF-reinforced biocomposites are not only a viable alternative to traditional materials but also offer a sustainable solution with enhanced mechanical and environmental benefits.

### Statement of originality

The authors declare that this manuscript is original, has not been published before and is not currently being considered for publication elsewhere. The authors confirm that the manuscript has been read and approved by all named authors and that there are no other persons who satisfied the criteria for authorship but are not listed. The authors further confirm that the order of authors listed in the manuscript has been approved by all of us. The authors understand that the corresponding author is the sole contact for the Editorial process. The corresponding author is responsible for communicating with the other authors about progress, submissions of revisions and final approval of proofs.

## Data Availability

The necessary data used in the manuscript are already present in the manuscript.

## Declarations

### Transparency statement

The authors affirm that this manuscript is an honest, accurate, and transparent account of the study being reported; that no important aspects of the study have been omitted; and that any discrepancies from the study as planned (and, if relevant, registered) have been explained.
